# Therapeutic strategies and promising vaccine for hepatitis C virus infection

**DOI:** 10.1002/iid3.977

**Published:** 2023-08-28

**Authors:** Adane Adugna

**Affiliations:** ^1^ Medical Microbiology, Medical Laboratory Sciences, College of Health Sciences Debre Markos University Debre Markos Ethiopia

**Keywords:** hepatitis C virus, promising vaccine, therapeutic strategies

## Abstract

Hepatitis C virus (HCV) infection is still a significant global health problem despite therapeutic advancements. Ribavirin and interferon therapy have been the sole available treatments for HCV infection for a number of years with low efficacy. Thus, currently, a number of therapeutic strategies are being used, including nanoparticles (NPs), micro‐RNAs such as small interfering RNA (siRNA), RNAi‐based gene silencing and antisense oligonucleotide‐based microRNA‐122, microRNA‐155, and short hairpin RNAs (shRNAs), and immunotherapeutic approaches such as anti‐programmed cell death 1(PD‐1), monoclonal antibodies (mAb or moAb), and monocyte‐derived dendritic cells (Mo‐DCs). Furthermore, direct‐acting antivirals (DAAs) and host‐targeting agents (HTA) were also the current therapeutic approaches with great efficacy. In spite of different clinical trials on HCV vaccine developments, nowadays there is no effective HCV vaccine in opposition to virus due to various challenges including genetic diversity, lack of immunocompetent small animal models, shortage of HCV vaccination testing alternatives, lack of an effective tissue culture method for replicating HCV, and inadequate knowledge regarding to immune responses against HCV infection. Nowadays, mRNA vaccine, recombinant viral vector, peptides vaccine, virus‐like particles, DNA vaccine, rational designed vaccine, and recombinant polyantigenic T‐cell‐based vaccine are novel promising candidates for HCV vaccine based on various clinical trials. This review summarizes the different therapeutic approaches and the advancements of vaccine candidates for HCV infection.

## 1 INTRODUCTION

Hepatitis C virus (HCV) is a significant global health issue and causes serious liver illnesses like hepatic cancer and above 350,000 illness die because of this cancer.^1^ Among HCV carriers, 75%–85% have a chance of having a chronic infection, whereas the remaining 20% show the virus spontaneously clear. Among chronic carriers, 5%–20% will develop cirrhosis. Cirrhosis patients have a 25% chance of developing hepatocellular carcinoma or end‐stage liver disease.^2^

The efficacy of the previous HCV infection therapy, pegylated interferon (IFN), is low and has major side effects. The creation of an HCV medication that is 100% efficient remains a challenge despite significant advancements in HCV treatment, such as the introduction of direct‐acting antivirals (DAAs) and host targeting agents (HTA).^3^ Various host amino acids that are critical in the viral life cycle and that control the host immune system and other cellular processes are the targets of HTAs.^4^ Moreover, micro‐RNAs, nano‐medicine, and immunotherapy are also the existing therapeutic strategies for patients with HCV infections.^5^, ^6^, ^7^

Hence, moving forward existing treatments and creating unused antiviral drugs with higher treatment adequacy and more favorable side‐effect profiles are of extraordinary clinical pertinence and importance.

Since RNA viruses like HCV lack a proofreading enzyme, errors will happen at the time of viral replication. Numerous genetic alterations cause a virus' genetic material to be inoperable as well as render it incapable of replicating. Others, however, continue to exist and are responsible for the enormous viral diversity that is unique to HCV, including its eight known genotypes and more than 100 subtypes. As the absence of a “proofreading” on RNA polymerase enzyme that performs replication causes the fast emergence of numerous but related quasispecies and blocks the development of vaccines against it and this could significantly affect the achievement of worldwide incidence‐reduction targets.^8^, ^9^, ^10^ Thus, this review will concentrate on the possible therapeutic approaches and promising vaccines for HCV.

## 2 THERAPEUTIC STRATEGIES FOR HCV INFECTION

### 2.1 DAAs for the treatment of HCV infection

Pegylated IFN‐alpha plus ribavirin, the standard treatment, does not successfully treat the infection in 30%–50% of cases because all patients do not experience a sustained response.^11^, ^12^ In comparison to the usual peginterferon/ribavirin treatment, the use of DAAs has significantly increased rates of persistent viral clearance ranging from 90% to 95%.^13^ Telaprevir and boceprevir, the initial two DAAs to be licensed to combat HCV infection, were created particularly to imitate the genotype 1 protease's native target and notably current authorized DAA combinations are glecaprevir/pibrentasvir and sofosbuvir/velpatasvir/voxilaprevir. These DAA regimens are pangenotypic.^14^ DAA has been demonstrated to be a safe treatment with few adverse effects.^15^

### 2.2 Host‐targeting agents (HTAs)

Via concentrating on host protein sequences that are maintained throughout the HCV life cycle, HTAs offer a fascinating viewpoint for new antiviral tactics towards HCV due to their significant genetic hurdle to the opposition, pan‐genotypic antiviral action, supplementary strategies of action to DAAs, and potential for cooperation with DAAs in clinical trials.^16^ Due to the exceedingly low mutational frequency that takes place inside host cells, HTAs offer a wide antiviral action with an extremely high genomic roadblock to drug tolerance.^17^

### 2.3 Micro‐RNAs as therapeutic strategies for HCV infection

Theoretically, a range of gene therapy‐based HCV defense mechanisms could be developed based on knowledge of the viral gene sequences. This intriguing strategy relies on the use of short RNA technology to stop viral enzyme activity and replication.^18^, ^19^ A group of tiny, genetically invariant noncoding RNAs known as miRNAs controls a number of crucial cellular functions. Since these compounds are so versatile, it was possible to anticipate with accuracy how they would impact the replication of HCV and its life cycle.^20^

RNA interference (RNAi)‐based gene silencing and antisense oligonucleotide‐based microRNA‐122 function suppression have demonstrated potential against HCV.^21^ The knockdown rates of viral gene silencers against different enveloped and nonstructural proteins, which varied from 70% to 93%, were similarly noteworthy.^22^ Because of its ability to cause cancer, microRNA (MiR‐155) is to blame for the development of cancer. Tumors occur at the sites because of the inflammation it produces. HCV‐related hepatocellular carcinoma has been linked with increased levels of MiR‐155 in liver cells. miR‐155 encourages cellular invasion, relocation, and proliferation during HCV infection.^23^

In reality, employing RNAi technology has allowed researchers to understand many of the crucial steps and participants in HCV entrance and replication. Short hairpin RNA (shRNA) is a potent method for inhibiting gene activity.^24^ DNA aptamers that target the HCV E1/E2 structural proteins have been shown to have a viral treatment effect.^25^

### 2.4 Nano‐medicine as a therapeutic strategy for HCV infection

For the administration of various medicinal products, such as anti‐HCV immunizing, testing, and therapeutic agents, nanomedicine has received a lot of interest. These substances were included within or connected to nanoparticles (NPs) made of various materials, including polymeric, lipid, and metallic NPs. To securely transport anti‐HCV medications to their target areas, there have been a number of obstacles to overcome. NPs were designed and customized to acquire special qualities to overcome these difficulties.^26^, ^27^, ^28^

Anti‐HCV medications were able to have an ongoing impact through pegylating NPs to limit drug absorption from the NPs framework or by magnetically reacting with carriers that had opposing electrical charges. Additionally, nanotechnology makes it simple to increase serum stability and safeguard anti‐HCV drugs.^29^, ^30^

### 2.5 Immunotherapeutic approach for HCV infection

#### 2.5.1 Anti‐programmed cell death 1 (PD‐1) as a therapeutic strategy for HCV infection

The exhaustion of CD8+ T lymphocytes that carry the inhibitory receptor for PD‐1 promotes the survival of the HCV (PD‐1). One characteristic of compromised immunity to malignancies and chronic infections is T‐cell depletion. Multiple inhibitory signaling receptors are constitutively expressed by exhausted CD8+ T lymphocytes, leading to loss of effector functions and decreased proliferative potential. For the purpose of restoring function to worn‐out T cells, blocking inhibitory signals with antibodies targeting receptors or their ligands is a viable therapeutic strategy. Improved in vitro proliferation of HCV‐specific T cells is achieved by blocking PD‐1 signaling. Antibodies against PD‐1 reduced the length of persistent infection and restored CD8+ T‐cell effector functions.^31^, ^32^

In patients with chronic hepatitis C, PD‐1 can be seen on the cell surface of CD8+ T lymphocytes that are specific for the HCV infection. On CD8+ T cells that are specifically targeting intact class I HCV epitopes without escape mutations to evade immune identification; this inhibitory receptor is expressed at its highest level. By antibody‐mediated inhibition of signaling through PD‐1 and other inhibitory receptors such as cytotoxic T lymphocyte antigen 4 (CTLA‐4), T‐cell Ig domain, and mucin domain 3, HCV antigen‐driven proliferation of CD8+ T cells was restored in cell culture (TIM‐3).^33^, ^34^

**Figure 1:**
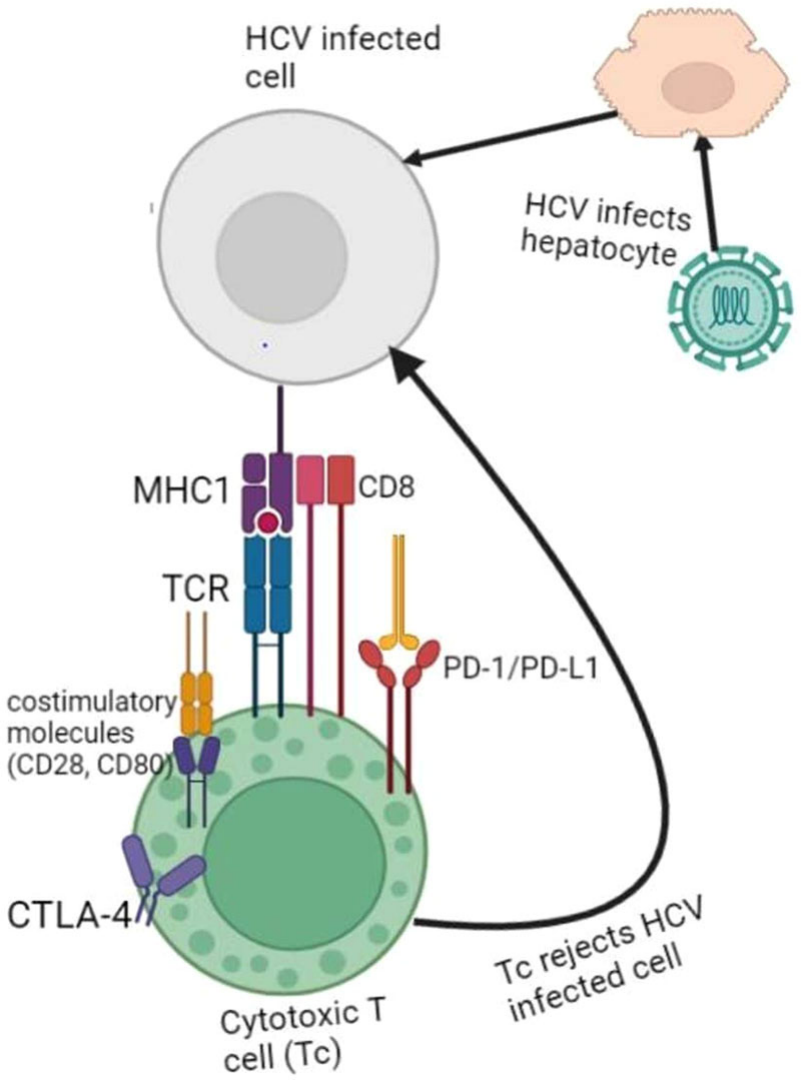
Host immunresponse induced by anti‐PD‐1 immunotherapy during chronic HCV infection. CTLA‐4, cytotoxic T lymphocyte antigen 4; HCV, hepatitis C virus; MHC, major histocompatibility complex; PD‐1/PD‐L1, programmed death‐ligand‐1; TCR, T‐cell receptor.

The immune inhibitory pathways targeting the (PD‐1)/programmed death‐ligand‐1 (PD‐L1) or the cytotoxic lymphocytes antigen proteins are suppressed by immune checkpoint inhibitor therapy, which has anticancer effects (CTLA‐4). The immune response is compromised in chronic hepatitis C patients due to the increase of PD‐1, PD‐L1, or CTLA‐4. Hepatocytes infected with viruses could be cleared by immunotherapy that blocks PD‐1, PD‐L1, or CTLA‐4 by reactivating the immune response to the viral antigen.^35^ The discovery that a protein known as cytotoxic T lymphocyte antigen‐4 (CTLA‐4) plays a significant inhibitory role in controlling T‐cell responses marked a turning point. Although CTLA‐4 is an intracellular protein, it translocates to the cell surface following T‐cell receptor engagement and a costimulatory signal through CD28. There, it competes with CD28 for binding to important costimulatory molecules (CD80, CD86) and mediates inhibitory signaling into the T cell, which stops both proliferation and activation^35^, ^36^ (Figure 1).

#### 2.5.2 Monoclonal antibodies (mAb or moAb) as a therapeutic approach for HCV infection

The administration of mAb is the alternative immunotherapy for HCV infection. Monospecific antibodies with the capacity to attach to a single epitope are known as mAb. These antibodies are produced by B cells, which are homogeneous hybrid cells and all clones of the same origin parent.^37^ For instance, mAb 2A5 attacks the HCV membrane and exhibits strong anti‐HCV neutralizing action in HCV‐infected patients.^38^

E1 and E2 HCV glycoproteins are the target of many HCV‐neutralizing antibodies. These antibodies block interactions between viral and host receptors. Innate immune system‐stimulating broad spectrum, anti‐HCV host‐targeting antivirals (HTAs) have been created. These anti‐HCV substances include agonists of the toll‐like receptors and anti‐scavenger receptor class B type I (anti‐SR‐BI). Some cellular components that are essential to the HCV lifecycle are inhibited as part of the action mechanism of this class of drugs.^39^, ^40^ Numerous broadly neutralizing anti‐HCV antibodies prevent CD81 from interacting with the HCV envelope glycoprotein E2.^41^

#### 2.5.3 Monocyte‐derived dendritic cells (Mo‐DCs)

It is also possible to treat HCV patients using Mo‐DCs that have been loaded with an HCV‐specific lipopeptide. To treat the patients, six lipopeptides made up of human leukocyte antigen‐A2 (HLA‐A2)‐restricted HCV‐specific cytotoxic T lymphocyte (CTL) epitopes were pulsed into the Mo‐DCs. Each of these lipopeptides was connected to a common Th epitope and the TLR2 agonist.^42^

#### 2.5.4 Promising vaccine for HCV

The difficulty of developing a vaccine is further complicated by the great genetic variability of the virus (since RNA‐dependent RNA polymerase lacks proofreading capability making it highly changeable). Due to this variability, HCV has greater than 100 subgroups and 8 identified genotypes.^10^ In addition to genetic diversity, shortage of HCV vaccination testing alternatives and suitable HCV experimental animals were other challenges to develop an effective HCV vaccine. Although not anymore utilized in HCV studies, chimpanzees were crucial in studying the immune response to the virus. Right now, it is only possible to make restricted evaluations regarding if vaccine‐induced adaptive immune responses will give protective immunity from HCV using immunocompetent small animal models.^43^

Furthermore, lack of an effective tissue culture method for replicating HCV, inadequate knowledge of immune responses that are protective against HCV, lack of evaluating neutralizing antibodies, and the ability of HCV to create multiple approaches to inhibit natural immune signaling and mask antigenic determinants were the previous challenges for the advancement of HCV vaccine with high protective efficacy.^44^

But recently, a number of encouraging strategies have been employed to create an HCV vaccine. Clinical trials must examine the protective effects of potential vaccination candidates in high incidence groups. In the future, a whole‐viral‐particle vaccination may be combined with methods that mainly stimulate T‐cell responses, such as vaccines based on viral vectors. An innovative new method for quickly testing potential HCV vaccination candidates is offered by mRNA vaccines. Infection resolution depends on a multi‐specific cellular immune response. For a vaccine to be effective, it may be necessary to induce high‐titer, long‐lasting, and cross‐reactive anti‐envelope antibodies as well as a robust multi‐specific cellular immune response that includes both helper and cytotoxic T cells.^45^, ^46^

An appealing target for a vaccination, the HCV envelope glycoproteins E1 and E2 are found at residues 192–746 of the polyprotein and are the targets of the humoral immune response.^47^ One potential peptide, which was recently described, was made up of overlapping peptides generated from the p7 protein. It stimulated antigen‐specific CD4+ T cells and cytotoxic CD8+ T cells that may target p7‐expressing hepatocytes in vivo.^48^

Autologous T cells from HCV‐naive individuals are stimulated to produce a variety of cytokines by dendritic cells (DCs) expressing HCV‐derived Core or NS3 antigens. Naive T cells can be naturally primed against certain antigens (Ags) by DCs. Human DCs were endogenously transfected with recombinant adenoviral vectors encoding HCV‐derived Core and NS3 proteins. These HCV Ags‐expressing DCs were used to activate and prime autologous T lymphocytes drawn from healthy, donors who were not infected. The HCV Core or NS3 Ag‐expressing DCs were able to activate T cells to release different cytokines and multiply in a way that was dependent on the HCV Ag. By using flow cytometry and antibody‐blocking tests, it was demonstrated that both CD4+ and CD8+ T cells responded to the HCV Core and NS3 in vitro.^49^

Since NS3 is an immunodominant protein, T cells that are reactive against NS3‐derived peptides play a crucial role in removing the virus from the body. Several inflammatory cytokine mRNAs were expressed by the HCV NS3 or core protein‐expressing DCs.^50^ Class II MHC molecules are found on antigen‐presenting cells and present antigenic peptides to T helper cells, but class I MHC molecules are found practically universally and only present intracellularly produced peptide fragments to CD8+ T cytotoxic cells. Accordingly, peptide vaccines based on this theory use short peptides found in the extracellular milieu and can attach to MHC class I or II molecules directly without going through the antigen processing steps. As a result, powerful immunogenic peptides created through chemical synthesis are being sought after as HCV vaccine candidates.^51^

Totally synthetic self‐adjuvanting lipopeptides with the dipalmitoyl‐*S*‐glyceryl cysteine lipid moiety, a ligand for Toll‐like receptor 2 (TLR2) on dendritic cells, have been used to successfully activate CD8+ T‐cell responses (DC). Additionally, it was discovered that human DC pulsed with lipopeptide secreted the pro‐inflammatory cytokine IL‐12p70 and were capable of triggering the production of IFN‐ by autologous CD8+ T cells isolated from a hepatitis C patient.^52^

Recombinant proteins, peptides, virus‐like particles (VLP), bare DNA, and recombinant viruses are among the novel vaccination options being investigated.^45^ The feasibility of production, the ease of altering DNA, and immune responses largely coming from many sources, like T helper cells and CTLs, as well as antibody responses, are all benefits of DNA immunization.^51^ Other molecular‐based vaccines include those that investigate DNA prime‐boost regimens, use multi‐epitope DNA, novel techniques like gene electrotransfer, and develop multigenotype vaccines with the addition of genetic adjuvants like perforin, granulocyte–monocyte colony‐stimulating factor (GM‐CSF), and interleukin‐12 (IL‐12).^53^, ^54^, ^55^, ^56^, ^57^, ^58^, ^59^

The other HCV vaccine candidate is alphavirus‐based DNA‐launched self‐amplifying RNA replicon (DREP) combined with a recombinant modified vaccinia virus Ankara (MVA) vector. With up to 70% of all CD8+ T cells being HCV specific in the adaptive phase, it was the most immunogenic combination and the first to show the beneficial effects of the translational enhancer in the enhancement of antigen‐specific T‐cell immune responses. It also elicited the highest levels of CD4+ and CD8+ HCV‐specific immune responses. Previously developed MVA‐HCV vaccine candidate contains nearly whole genotype 1a HCV genome (strain H77). A synthetic early/late promoter regulates the transcription of the HCV core, E1, E2, p7, NS2, NS3, NS4A, NS4B, and NS5A proteins, as well as a portion of NS5B, by MVA‐HCV.^60^

**Figure 2:**
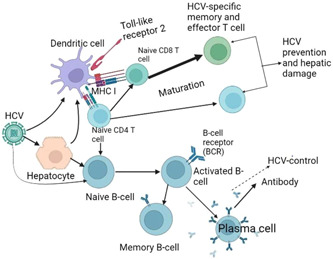
Induced host immunity by hepatitis C virus (HCV)‐vaccine candidates.

A vaccine vector with a strong immunogenicity and a stellar safety record is modified vaccinia Ankara (MVA). MVA is especially efficient as a boosting vector, expanding and amplifying the scope of pre‐existing T‐cell responses.^46^ Chimpanzees with core‐E1‐E2‐ and NS3‐encoding recombinant MVA and HCV DNA plasmids that encode these proteins were infected. Significant CD8+ T‐cell responses against HCV, high HCV‐specific antibody titers, and strong Th1‐ and Th2‐cytokine responses were all brought on by this DNA primed‐MVA boosted immunization.^61^ Clinical trials using TG4040, a recombinant polyantigenic T‐cell vaccine based on MVA, have shown promise as a therapeutic vaccine. The HCV NS3, NS4, and NS5B proteins are encoded by TG4040. It was initially examined in HLA31 class I transgenic mice, and it was discovered to create powerful, enduring cross‐reactive CD8+ and CD4+ T lymphocytes specific for HCV (Figure 2).^62^

Another vaccine candidate for HCV was rationally designed vaccine. By examining the virus's antibody range in the early phases of vaccine rational design research, neutralizing antibodies linked to certain virus clearance can be identified. Then, particular antigens can be deliberately designed to encourage the formation of such antibodies. For instance, the rational design of a particular epitope in E2 can result in the production of the potent antiviral antibody HC33.1.^63^ Recent studies have successfully developed an anti‐idiotypic stimulant of the AP33 epitope region. An immunogen was used to generate an antibody with the same recognition site and position as AP33 in a mouse model, and the antibody was capable of preventing HCV infection.^64^

## 3 CONCLUSIONS

In conclusion, different therapeutic approaches are being investigated for patients with HCV infection including DAAs, HTA, NPs, micro‐RNAs, and immunotherapies. Synthetic peptides, mRNA‐based vaccines, DNA‐based vaccines, recombinant viral vector vaccines, VLP, recombinant polyantigenic T‐cell‐based vaccine, and rationally designed vaccine are all now being tested in clinical trials for the HCV vaccine and a potent HCV vaccine candidate should successfully elicit lifetime protection in humans by eradicating the virus and producing broadly cross‐receptive CD4 and CD8 T cells as well as potently neutralizing antibodies.

## AUTHOR CONTRIBUTIONS

Adane Adugna is involved in conceptualization of this review, prepared manuscript draft and writing‐up, manuscript approval and validation, manuscript editing, language editing, and design, and as this author is the only author of this manuscript, involved in all aspects of this paper.

## CONFLICT OF INTEREST STATEMENT

The author declares no conflict of interest.
